# Dietary Acid Load Is Positively Associated With Risk of Gestational Diabetes Mellitus in a Prospective Cohort of Chinese Pregnant Women

**DOI:** 10.3389/fnut.2022.892698

**Published:** 2022-05-26

**Authors:** Rui Zhao, Leilei Zhou, Gang Lei, Shanshan Wang, Yan Li, Xuefeng Yang, Guoping Xiong, Liping Hao

**Affiliations:** ^1^Department of Nutrition and Food Hygiene, Hubei Key Laboratory of Food Nutrition and Safety and the Ministry of Education (MOE) Key Laboratory of Environment and Health, School of Public Health, Tongji Medical College, Huazhong University of Science and Technology, Wuhan, China; ^2^The Central Hospital of Wuhan, Wuhan, China

**Keywords:** dietary acid load, gestational diabetes mellitus (GDM), potential renal acid load (PRAL), net endogenous acid production (NEAP), animal protein to potassium ratio (A:P ratio), cohort

## Abstract

**Background:**

Growing evidence suggests that dietary acid load plays an important role in the development of type 2 diabetes. However, prospective studies on the relationship between dietary acid load and gestational diabetes mellitus (GDM) are limited in the pregnant population. This study aimed to investigate the effect of dietary acid load during early pregnancy on the risk of GDM in Chinese pregnant women.

**Methods:**

A total of 1,327 pregnant women were enrolled from an ongoing prospective study of the Tongji Birth cohort (TJBC) in Wuhan, China. Dietary intake was assessed before 20 weeks using a 74-item semiquantitative food frequency questionnaire (FFQ). The dietary acid load was estimated using potential renal acid load (PRAL), net endogenous acid production (NEAP), and animal protein to potassium ratio (A:P ratio). A 75g 2-h oral glucose tolerance test (OGTT) was performed at 24-28 gestational weeks to diagnose GDM.

**Results:**

The mean (standard deviation) values for PRAL score, NEAP score, and A:P ratio were 0.8 ± 11.3 mEq/day, 45.3 ± 16.5 mEq/day, and 9.8 ± 6.0, respectively. There was a significant positive correlation of dietary acid load with the intake of red meat, poultry, fish, and eggs, and a negative correlation with the intake of vegetables, fruits, nuts, and legumes (all *P* < 0.05). Compared to the lowest tertile, the highest tertile of dietary acid load, including PRAL score (odds ratio [OR]: 2.26, 95% confidence interval [CI] = 1.38–3.71, *P*-trend = 0.002), NEAP score (OR: 2.02, 95% CI = 1.25–3.27, *P*-trend = 0.009), and A:P ratio (2.08, 95% CI = 1.30–3.31, *P*-trend = 0.005), significantly increased the risk of GDM. In addition, the dietary acid load was also significantly associated with an increase in 1-h and 2-h post-load blood glucose concentrations (all *P*-trend < 0.05).

**Conclusion:**

We found a significant positive association between dietary acid load during early pregnancy and the risk of GDM in a Chinese population, suggesting that the reduction of food sources of dietary acid load may be an effective strategy for preventing the risk of GDM.

## Introduction

Gestational diabetes mellitus (GDM) is defined as glucose intolerance that is new onset or first recognized during pregnancy (1). As one of the most common complications of pregnancy, GDM affects approximately 5.8-20.7% of pregnant women worldwide (2, 3). A systematic review and meta-analysis showed that the prevalence of GDM in Chinese pregnant women was 14.8% (95% confidence interval [CI]: 12.8-16.7%) (4). In the short term, pregnant women with GDM are at higher risk of preterm birth, macrosomia, and cesarean section (5, 6). In addition, it can also have long-term effects, leading to overweight (7) and neurodevelopmental disorders (8) in the offspring and a higher risk of type 2 diabetes in the mothers (9). Several risk factors for the development of GDM have been identified in previous studies, such as maternal age, family history of diabetes, pre-pregnancy body mass index (pre-pregnancy BMI), gestational weight gain, and multiple births (10, 11). The identification of modifiable risk factors that contribute to the prevention of GDM is of great importance in promoting the health of mothers and offspring.

In recent years, the role of dietary acid load in the etiology of insulin resistance and type 2 diabetes has attracted increasing attention (12–17). It has been suggested that acid-base disturbance may contribute to the development of insulin resistance (18, 19). Randomized controlled trials have demonstrated that a short-term vegan dietary intervention is effective in reducing dietary acid load and raising 24-h urine pH in healthy individuals (20, 21). Similarly, observational studies have shown that Western dietary patterns (high intake of acidogenic foods including animal products, and low intake of alkalizing foods including fruits and vegetables) might lead to excessive production of endogenous acids and dietary acid-base imbalances, which in turn might contribute to the development of type 2 diabetes (16, 22). Currently, there are three main indicators for evaluating dietary acid load produced by overall diet, including potential renal acid load (PRAL), net endogenous acid production (NEAP), and animal protein to potassium ratio (A:P ratio). A recent meta-analysis of observational studies showed that higher dietary acid load levels, particularly PRAL scores, were associated with an increased risk of type 2 diabetes (17). The results of a longitudinal study suggested that higher diet-dependent acid load, both PRAL and NEAP scores, is positively associated with the development of insulin resistance (12). However, prospective evidence for the effect of dietary acid load on GDM risk is limited. Only one case-control study in Iran has examined the association between dietary acid load and GDM risk, showing that higher dietary acid load was associated with greater odds of GDM (23). Given the wide variation in dietary habits across regions, it is valuable to provide additional data from the Chinese Population to improve the generalizability of the findings.

Therefore, this study aimed to prospectively evaluate the relationship between dietary acid load in early pregnancy and the risk of GDM in Chinese pregnant women using the PRAL score, NEAP score, and A:P ratio.

## Materials and Methods

### Study Population

**FIGURE 1:**
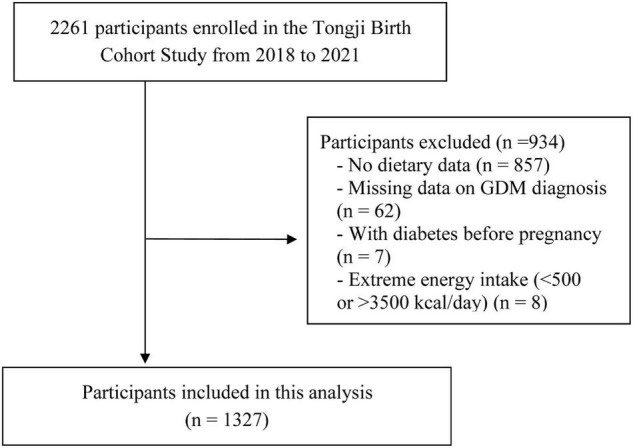
Flow chart for the selection of subjects included in the analysis.

Data was used from the prospective cohort study of Tongji Birth Cohort (TJBC) in Wuhan, China. The TJBC study was established in 2018 to assess the role of nutritional status and environmental exposures in maternal and child health. Pregnant women with a single pregnancy, gestational age < 20 weeks, planning to deliver at a participating hospital, and agreeing to complete a face-to-face questionnaire were included in the study (*n* = 2261). For the present analyses, we excluded participants with pre-pregnancy diabetes (*n* = 7), no dietary data on early pregnancy (*n* = 857), extreme energy intake (< 500 kcal/day or > 3500 kcal/day) (*n* = 8), and lack of GDM diagnosis (*n* = 62), with a total of 1,327 participants finally being included (Figure 1). Ethical approval was obtained from the Ethics Committee of Tongji Medical College of Huazhong University of Science and Technology, and all participants provided written informed consent before enrollment.

### Dietary Assessment

Dietary intake was assessed through face-to-face interviews using a 74-item semiquantitative food frequency questionnaire (FFQ), which has been proven in the previous study to be a reasonable tool for assessing nutrient and food intakes of pregnant women in China (24). The description of FFQ has been described in detail in the previous study (25). In brief, pregnant women were asked about the frequency and amount of the 74 food items consumed over the past four weeks. The frequency of food intake ranged from “less than once a month” to “more than three times a day” among the 13 frequency options. Trained dietitians used a color food photography atlas containing different portion sizes of all foods and food models representing the standard portions to make the estimation more accurate. The daily intake of energy and nutrients was calculated by FFQ based on the Chinese Food Composition Tables (26). Food and nutrient intakes were adjusted according to the energy residual method (27).

### Dietary Acid Load

In this study, we calculated the dietary acid load through three different measures: potential renal acid load (PRAL) (28), net endogenous acid production (NEAP) (29), and animal protein-to-potassium ratio (A:P ratio) (30).

The equations are as follows:

(1) PRAL (mEq/day) = (0.4888 × protein (g/day)) + (0.0366 × phosphorus (mg/day)) − (0.0205 × potassium (mg/day)) − (0.0263 × magnesium (mg/day))−(0.0125 × calcium (mg/day));
(2) NEAP (mEq/day) = 54.5 × protein intake (g/day)/ potassium intake (mEq/day) − 10.2;
(3) A:P ratio = animal protein (g/day)/potassium (g/day).

### Outcome Definitions

A 75 g 2-h oral glucose tolerance test (OGTT) was performed for all pregnant women at 24-28 gestational weeks after at least 8-h of fasting. Fasting blood glucose (FBG), 1-h post-load blood glucose (PBG), and 2-h PBG levels were collected from medical records. According to the criteria established by the International Association of the Diabetes and Pregnancy Study Groups, subjects were diagnosed with GDM if they met any of the following criteria: FBG ≥ 5.1 mmol/L; 1-h PBG ≥ 10.0 mmol/L; or 2-h PBG ≥ 8.5 mmol/L (31).

### Other Variables

Information on covariates was obtained through a structured questionnaire completed at enrolment, including maternal age, education level, gravidity, parity, personal and family history of diabetes, and lifestyle habits before pregnancy such as smoking status, alcohol intake, and physical activity. Alcohol consumers (or smokers) were defined as drinking (or smoking) more than one time a week before pregnancy. Participants were considered to have regular physical activity if they reported physical activity at least once a week before pregnancy. We also collected data on anthropometric measurements, including maternal height and pre-pregnancy weight. Pre-pregnancy BMI was calculated using self-reported pre-pregnancy weight (kg) divided by height^2^ (m^2^).

### Statistical Analyses

Data are presented as mean (standard deviation [SD]) for continuous variables and n (%) for categorical variables. One-way analysis of variance and the Chi-squared test were used to compare continuous and categorical variables, respectively. The PRAL score, NEAP score, and A:P ratio were categorized in tertiles, with the lowest tertile as the reference group. Multivariable logistic regression analyses were used to assess the associations between dietary acid load levels in early pregnancy and risk of GDM, with the results expressed as odds ratios (ORs) and 95% CIs. In order to test the significance of linear trends across tertiles, the median value of each tertile of dietary acid load measures was considered to be a continuous variable. Generalized linear models were conducted to examine the association of dietary acid load levels with FBG, 1-h PBG, and 2-h PBG, and the results were presented as coefficients (β) with 95% CIs. All potential confounders in the multivariable models were chosen based on both biological and statistical considerations (changed main effect estimates > 10%). Multivariate models were as followed: (1) model 1 was the crude model; (2) model 2 adjusted for maternal age (continuous), pre-pregnancy BMI (continuous), education years (≤ 12, 13–15, ≥ 16 years), primiparity (yes/no), smoking status before pregnancy (yes/no), alcohol intake before pregnancy (yes/no), regular physical activity before pregnancy (yes/no), and family history of diabetes (yes/no); (3) model 3 further adjusted for energy-adjusted nutrient intake (i.e., carbohydrate, dietary fiber, cholesterol, vitamin A, vitamin C, vitamin E, saturated fatty acids (SFAs), and Monounsaturated fatty acids (MUFAs)).

To evaluate the potential modification effect, stratified analyses were conducted according to the median value of maternal age (< 29.2 or ≥ 29.2 years), pre-pregnancy BMI (< 20.5 or ≥ 20.5 kg/m^2^), primiparity (yes or no), gravidity (yes or no), regular physical activity (yes or no), and family history of diabetes (yes or no). The likelihood ratio tests were used to assess the interactions between stratified variables and freshwater fish intake. In addition, we performed different sensitivity analyses to assess the stability of the study results. First, we excluded participants who were over 30 years old at the time of pregnancy. Second, we excluded participants with abnormal pre-pregnancy BMI (< 18.5 or ≥ 24 kg/m^2^). Third, we separately excluded participants with smoking or alcohol consumption habits before pregnancy. All analyses were performed using statistical packages R (The R Foundation; v. 3.4.3)^1^ and Empower(R) (X&Y Solutions Inc.)^2^. We considered *P* < 0.05 in the two-sided test as significant.

## Results

### Characteristics of Participants

A total of 1,327 subjects were included in the present study (Figure 1). The mean (SD) values for PRAL score, NEAP score, and A:P ratio in the study population were 0.8 ± 11.3 mEq/day, 45.3 ± 16.5 mEq/day, and 9.8 ± 6.0, respectively. Table 1 shows the characteristics of study participants by tertiles of the PRAL score distribution. Compared to those with the lowest tertile of PRAL scores (< −3.2 mEq/day), individuals with the highest tertile of PRAL scores (≥ 5.3 mEq/day) were more likely to be multiparous and to have a family history of diabetes. For specific food groups, participants with higher PRAL scores consumed more grains and animal products (red meat, poultry, fish, eggs) and fewer vegetables, fruit, and legumes than participants with lower PRAL scores. In addition, they also had higher intakes of protein, cholesterol, SFAs, and MUFAs, and lower intakes of carbohydrates, dietary fiber, vitamin A, vitamin C, vitamin E, potassium, calcium, and magnesium.

**TABLE 1:** Characteristics of study participants according to tertiles of the PRAL score^a^.

Variables	Overall (*n* = 1327)	Tertiles of PRAL score (mEq/day)			*P* value^b^
		T1 (*n* = 442)	T2 (*n* = 442)	T3 (*n* = 443)	
**Maternal Characteristics**					
Maternal age (years)	29.5 ± 3.3	29.4 ± 3.6	29.5 ± 3.1	29.7 ± 3.4	0.365
Pre-pregnancy BMI (kg/m^2^)	21.1 ± 3.1	20.9 ± 2.8	21.0 ± 3.2	21.3 ± 3.2	0.170
Education (years), n (%)					0.919
≤ 12	304 (22.9%)	105 (23.8%)	97 (21.9%)	102 (23.0%)	
13–15	427 (32.2%)	146 (33.0%)	139 (31.4%)	142 (32.1%)	
≥ 16	590 (44.5%)	189 (42.8%)	203 (45.9%)	198 (44.7%)	
Income (CNY/month), n (%)					0.193
≤ 4999	189 (14.2%)	60 (13.6%)	70 (15.8%)	59 (13.3%)	
5000–9999	734 (55.3%)	240 (54.3%)	242 (54.8%)	252 (56.9%)	
≥ 10000	376 (28.3%)	128 (29.0%)	127 (28.7%)	121 (27.3%)	
Gravidity (times), n (%)					0.451
1	789 (59.5%)	266 (60.2%)	270 (61.1%)	253 (57.1%)	
≥ 2	538 (40.5%)	176 (39.8%)	172 (38.9%)	190 (42.9%)	
Primiparity (yes), n (%)	1069 (80.6%)	362 (81.9%)	366 (82.8%)	341 (77.0%)	0.062
Alcohol intake (yes), n (%)	29 (2.2%)	8 (1.8%)	8 (1.8%)	13 (2.9%)	0.418
Smoking status (yes), n (%)	36 (2.7%)	11 (2.5%)	12 (2.7%)	13 (2.9%)	0.920
Regular physical activity (yes), n (%)	503 (37.9%)	177 (40.0%)	167 (37.8%)	159 (35.9%)	0.444
Family history of diabetes (yes), n (%)	150 (11.3%)	31 (7.0%)	56 (12.7%)	63 (14.2%)	0.010
GDM, n (%)	217 (16.4%)	53 (12.0%)	82 (18.6%)	82 (18.5%)	0.010
**Food intake^c^**					
Grains (g/day)	245.2 ± 58.9	223.8 ± 58.7	245.3 ± 54.9	266.2 ± 55.3	< 0.001
Vegetables (g/day)	309.8 ± 139.0	376.0 ± 155.7	309.6 ± 120.2	244.0 ± 103.2	< 0.001
Fruits (g/day)	485.7 ± 219.3	649.0 ± 229.4	453.0 ± 152.5	355.3 ± 154.0	< 0.001
Red meats (g/day)	31.3 ± 34.6	18.6 ± 18.1	27.8 ± 21.2	47.4 ± 48.9	< 0.001
Poultry (g/day)	7.4 ± 11.7	5.3 ± 8.6	7.4 ± 10.8	9.5 ± 14.7	< 0.001
Fish (g/day)	27.7 ± 27.5	22.8 ± 21.9	26.3 ± 24.7	33.9 ± 33.5	< 0.001
Eggs (g/day)	31.7 ± 23.9	28.1 ± 24.8	30.6 ± 21.5	36.4 ± 24.6	< 0.001
Dairy products (ml/day)	165.2 ± 136.0	167.2 ± 144.0	170.2 ± 131.8	158.2 ± 131.8	0.394
Nuts (g/day)	13.3 ± 13.2	14.4 ± 14.5	13.0 ± 12.7	12.6 ± 12.2	0.117
Legumes (g/day)	7.9 ± 8.2	8.1 ± 6.8	8.1 ± 9.0	7.5 ± 8.6	0.473
**Nutrient intake^c^**					
Energy (kcal/day)	1899.9 ± 492.8	1915.5 ± 516.0	1923.1 ± 451.6	1861.1 ± 507.1	0.125
Protein (g/day)	57.7 ± 14.4	51.1 ± 10.0	55.9 ± 8.4	66.1 ± 18.2	< 0.001
Animal protein (g/day)	21.8 ± 12.9	16.5 ± 8.1	20.4 ± 8.9	28.4 ± 16.7	< 0.001
Plant protein (g/day)	35.9 ± 7.3	34.4 ± 7.0	35.3 ± 5.8	38.1 ± 8.4	< 0.001
Fat (g/day)	68.8 ± 15.5	67.3 ± 15.1	70.1 ± 15.9	69.2 ± 15.6	0.022
Carbohydrates (g/day)	289.9 ± 35.8	296.8 ± 34.2	287.3 ± 36.4	285.5 ± 35.8	< 0.001
Dietary fiber (g/day)	14.6 ± 3.5	16.8 ± 3.6	14.3 ± 2.8	12.6 ± 2.6	< 0.001
Cholesterol (mg/day)	296.0 ± 164.9	260.9 ± 165.6	286.4 ± 149.0	340.7 ± 169.6	< 0.001
Vitamin A (ugRAE/day)	781.3 ± 331.9	922.7 ± 355.3	764.7 ± 302.0	656.7 ± 278.6	< 0.001
Vitamin C (mg/day)	186.7 ± 68.5	238.9 ± 70.8	179.9 ± 47.4	141.6 ± 45.0	< 0.001
Vitamin E (mg/day)	39.6 ± 12.7	42.3 ± 13.0	39.9 ± 13.1	36.8 ± 11.4	< 0.001
Dietary SFAs (g/day)	14.8 ± 4.2	13.9 ± 3.9	15.1 ± 4.0	15.5 ± 4.5	< 0.001
Dietary MUFAs (g/day)	22.7 ± 8.1	21.7 ± 8.0	23.0 ± 8.1	23.4 ± 8.1	0.005
Dietary PUFAs (g/day)	23.1 ± 8.7	23.3 ± 9.0	23.5 ± 9.0	22.5 ± 8.1	0.156
Sodium (mg/day)	385.4 ± 155.8	391.6 ± 136.6	381.5 ± 145.9	383.2 ± 181.3	0.589
Potassium (g/day)	2.3 ± 0.4	2.6 ± 0.4	2.2 ± 0.3	2.0 ± 0.3	< 0.001
Calcium (mg/day)	536.3 ± 166.8	576.1 ± 170.3	535.2 ± 161.6	497.7 ± 159.5	< 0.001
Magnesium (mg/day)	314.7 ± 46.2	341.0 ± 45.7	310.0 ± 40.4	293.0 ± 38.6	< 0.001
Phosphorus (mg/day)	937.3 ± 119.7	936.6 ± 116.8	931.1 ± 120.6	944.1 ± 121.7	0.266
PRAL score (mEq/day)	0.8 ± 11.3	−11.0 ± 7.1	1.1 ± 2.4	12.3 ± 7.7	< 0.001
NEAP score (mEq/day)	45.3 ± 16.5	31.2 ± 6.9	43.4 ± 4.0	61.1 ± 17.3	< 0.001
A:P ratio	9.8 ± 6.0	6.2 ± 2.7	9.0 ± 3.2	14.1 ± 7.7	< 0.001

*^a^Values are expressed as mean ± standard deviation or n (%).*

*^b^P value was obtained using the chi-square test for categorical variables and ANOVA tests for continuous variables.*

*^c^Energy-adjusted using the residual method. A:P ratio, animal protein to potassium ratio; pre-pregnancy BMI, pre-pregnancy body mass index; Eq, equivalent; GDM, gestational diabetes mellitus; MUFAs, Monounsaturated fatty acids; NEAP, net endogenous acid production; PRAL, potential renal acid load; PUFAs, Polyunsaturated fatty acids; SFAs, saturated fatty acids; T, tertile.*

### Correlation Between Dietary Acid Load and Food Intake

Table 2 shows the Pearson correlation coefficients between dietary acid load scores and food intake. There were statistically significant positive correlations between intake of most animal foods (red meats, poultry, fish, and eggs) and dietary acid load scores (all *P* < 0.05), except for dairy products. Regarding plant foods, we observed significant negative correlations of vegetables, fruits, nuts, and legumes intake with dietary acid load (all *P* < 0.05). However, grains intake was positively correlated with PRAL and NEAP scores, while it was negatively correlated with the A:P ratio.

**TABLE 2:** Pearson correlations between food group intake and three dietary acid load measures^a^.

Food group^b^	Dietary acid load					
	PRAL score	*P* value	NEAP score	*P* value	A:P ratio	*P* value
Grains (g/day)	0.177	< 0.001	0.278	< 0.001	−0.100	< 0.001
Vegetables (g/day)	−0.223	< 0.001	−0.305	< 0.001	−0.197	< 0.001
Fruits (g/day)	−0.261	< 0.001	−0.452	< 0.001	−0.263	< 0.001
Nuts (g/day)	−0.059	0.132	−0.090	0.002	−0.047	0.104
Legumes (g/day)	−0.071	0.065	−0.059	0.040	−0.102	< 0.001
Total meats g/day)	0.140	< 0.001	0.246	< 0.001	0.608	< 0.001
Red meats (g/day)	0.280	< 0.001	0.453	< 0.001	0.571	< 0.001
Poultry (g/day)	0.135	0.002	0.214	< 0.001	0.355	< 0.001
Fish (g/day)	0.086	0.024	0.121	< 0.001	0.360	< 0.001
Eggs (g/day)	0.055	0.144	0.078	0.007	0.253	< 0.001
Dairy products (ml/day)	−0.024	0.533	−0.017	0.546	0.281	< 0.001

*^a^Food group intakes and three dietary acid load measures were log10-transformed to improve normality.*

*^b^Energy-adjusted using the residual method. A:P ratio, animal protein to potassium ratio; NEAP, net endogenous acid production; PRAL, potential renal acid load.*

### Association Between Maternal Dietary Acid Load and GDM Risk

The associations between indices of dietary acid load and GDM risk were shown in Table 3. In the multivariable models, PRAL score, NEAP score, and A:P ratio were all associated with an increased risk of GDM after adjusting for covariates of maternal age, pre-pregnancy BMI, education, primiparity, smoking status, alcohol intake, regular physical activity, family history of diabetes, and other dietary factors. The multivariable-adjusted ORs (95% CIs) of GDM for the lowest to the highest tertiles of PRAL score were 1.00 (reference), 2.06 (1.35, 3.15), and 2.26 (1.38, 3.71) (*P*-trend = 0.002). Similar findings were found for the NEAP score (OR for T3 vs. T1: 2.02, 95% CI: 1.25–3.27; T2 vs. T1: 2.05, 95% CI: 1.36–3.10; *P*-trend = 0.009). In addition, those in the highest tertile of the A:P ratio had a 108% higher risk of GDM than those in the lowest tertile after controlling for potential covariates (OR: 2.08, 95% CI: 1.30–3.31, *P*-trend = 0.005).

**TABLE 3:** Associations between maternal dietary acid load and risk of GDM.

Variables	OR (95% CI)				
	Median (IQR)	Cases/N	Crude model	Multivariate model I^a^	Multivariate model II^b^
**PRAL score**					
T1	−8.91 (−13.79–6.00)	53/442	1.00	1.00	1.00
T2	1.22 (−0.79-3.23)	82/442	1.67 (1.15, 2.43)	1.69 (1.14, 2.50)	2.06 (1.35, 3.15)
T3	10.15 (7.30-14.48)	82/443	1.67 (1.15, 2.42)	1.64 (1.11, 2.43)	2.26 (1.38, 3.71)
*P*-trend^c^			0.007	0.015	0.002
**NEAP score**					
T1	32.74 (27.73-35.92)	52/442	1.00	1.00	1.00
T2	43.47 (40.84-46.31)	86/442	1.81 (1.25, 2.63)	1.78 (1.21, 2.63)	2.05 (1.36, 3.10)
T3	55.62 (51.54-64.79)	79/443	1.63 (1.12, 2.38)	1.63 (1.10, 2.42)	2.02 (1.25, 3.27)
*P*-trend^c^			0.019	0.025	0.009
**A:P ratio**					
T1	5.32 (3.80-6.29)	50/442	1.00	1.00	1.00
T2	8.85 (8.11-9.62)	81/442	1.76 (1.20, 2.57)	1.65 (1.11, 2.46)	1.85 (1.23, 2.79)
T3	13.75 (12.02-16.34)	86/443	1.89 (1.30, 2.75)	1.77 (1.19, 2.62)	2.08 (1.30, 3.31)
*P*-trend^c^			0.002	0.008	0.005

*^a^Multivariate model I was adjusted for maternal age, pre-pregnancy BMI, education, primiparity, smoking status, alcohol intake, regular physical activity, and family history of diabetes.*

*^b^Multivariate model II was further adjusted for intake of carbohydrate, dietary fiber, cholesterol, vitamin A, vitamin C, vitamin E, SFAs, and MUFAs.*

*^c^Tests for linear trend were conducted by using the median value for each tertile and treating it as a continuous variable in the logistic regression. A:P ratio, animal protein to potassium ratio; GDM, gestational diabetes mellitus; IQR, interquartile range; NEAP, net endogenous acid production; OR, odds ratio; PRAL, potential renal acid load, T, tertile.*

### Association Between Maternal Dietary Acid Load and Blood Glucose Concentrations

In the crude model, the highest tertile of dietary acid load (PRAL score, NEAP score, and A:P ratio) in early pregnancy was associated with an increase in FBG, 1-h PBG, and 2-h PBG compared to the lowest tertile. After controlling for potential covariates, we found that women in the highest tertile of the PRAL score significantly increased FBG by 0.09 mmol/L (95% CI: 0.02, 0.17, *P-*trend = 0.017), 1-h PBG by 0.50 mmol/L (95% CI: 0.19, 0.81, *P-*trend = 0.002) and 2-h PBG by 0.54 mmol/L (95% CI: 0.28, 0.80, *P-*trend < 0.001), respectively, compared to women in the lowest tertile. Similarly, we identified the significant positive relationships of NEAP score and A:P ratio with 1-h PBG (β = 0.47, 95% CI: 0.17, 0.77, *P-*trend = 0.003 for NEAP; β = 0.31, 95% CI: 0.01, 0.61, *P-*trend = 0.044 for A:P ratio) and 2-h PBG (β = 0.43, 95% CI: 0.18, 0.69, *P-*trend = 0.001 for NEAP; β = 0.28, 95% CI: 0.03, 0.53, *P-*trend = 0.041 for A:P ratio) when the highest tertile compared to the lowest tertile (Table 4).

**TABLE 4:** Associations between maternal dietary acid load and blood glucose levels.

Variables	β (95% CI), mmol/L		
	Crude model	Multivariate model I^a^	Multivariate model II^b^
**PRAL score**			
**FBG**			
T1	0.00	0.00	0.00
T2	0.08 (0.02, 0.14)	0.06 (0.00, 0.12)	0.07 (0.01, 0.14)
T3	0.10 (0.04, 0.16)	0.08 (0.02, 0.13)	0.09 (0.02, 0.17)
*P*-trend^c^	0.001	0.009	0.017
**1-h PBG**			
T1	0.00	0.00	0.00
T2	0.21 (−0.04, 0.46)	0.09 (−0.15, 0.33)	0.26 (−0.01, 0.53)
T3	0.37 (0.12, 0.61)	0.23 (0.00, 0.47)	0.50 (0.19, 0.81)
*P*-trend^c^	0.003	0.051	0.002
**2-h PBG**			
T1	0.00	0.00	0.00
T2	0.29 (0.09, 0.50)	0.18 (−0.02, 0.38)	0.40 (0.18, 0.63)
T3	0.33 (0.13, 0.53)	0.23 (0.03, 0.42)	0.54 (0.28, 0.80)
*P*-trend^c^	0.001	0.022	< 0.001
**NEAP score**			
**FBG**			
T1	0.00	0.00	0.00
T2	0.05 (−0.01, 0.12)	0.03 (−0.03, 0.09)	0.04 (−0.02, 0.11)
T3	0.08 (0.02, 0.14)	0.06 (−0.00, 0.11)	0.06 (−0.01, 0.14)
*P*-trend^c^	0.010	0.053	0.113
**1-h PBG**			
T1	0.00	0.00	0.00
T2	0.27 (0.02, 0.52)	0.15 (−0.09, 0.39)	0.31 (0.04, 0.57)
T3	0.36 (0.11, 0.60)	0.25 (0.02, 0.49)	0.47 (0.17, 0.77)
*P*-trend^c^	0.004	0.034	0.003
**2-h PBG**			
T1	0.00	0.00	0.00
T2	0.36 (0.15, 0.56)	0.25 (0.05, 0.45)	0.43 (0.20, 0.65)
T3	0.29 (0.08, 0.49)	0.21 (0.02, 0.41)	0.43 (0.18, 0.69)
*P*-trend^c^	0.008	0.039	0.001
**A:P ratio**			
**FBG**			
T1	0.00	0.00	0.00
T2	0.08 (0.02, 0.14)	0.05 (−0.01, 0.11)	0.06 (−0.01, 0.12)
T3	0.09 (0.03, 0.15)	0.06 (0.00, 0.12)	0.05 (−0.03, 0.12)
*P*-trend^c^	0.006	0.043	0.309
**1-h PBG**			
T1	0.00	0.00	0.00
T2	0.13 (−0.12, 0.38)	0.01 (−0.23, 0.25)	0.15 (−0.12, 0.41)
T3	0.27 (0.03, 0.52)	0.13 (−0.10, 0.37)	0.31 (0.01, 0.61)
*P*-trend^c^	0.031	0.245	0.044
**2-h PBG**			
T1	0.00	0.00	0.00
T2	0.18 (−0.03, 0.39)	0.09 (−0.11, 0.29)	0.23 (0.01, 0.45)
T3	0.20 (−0.01, 0.40)	0.09 (−0.11, 0.29)	0.28 (0.03, 0.53)
*P*-trend^c^	0.075	0.411	0.041

*^a^Multivariate model I was adjusted for maternal age, pre-pregnancy BMI, education, primiparity, smoking status, alcohol intake, regular physical activity, and family history of diabetes.*

*^b^Multivariate model II was further adjusted for intake of carbohydrate, dietary fiber, cholesterol, vitamin A, vitamin C, vitamin E, SFAs, and MUFAs.*

*^c^Tests for linear trend were conducted by using the median value for each tertile and treating it as a continuous variable in the logistic regression. A:P ratio, animal protein to potassium ratio; FBG, fasting blood glucose; NEAP, net endogenous acid production; PBG, post-load blood glucose; PRAL, potential renal acid load, T, tertile.*

### Subgroup and Sensitivity Analyses

To assess whether other confounding factors modified the association between dietary acid load and risk of GDM, we performed stratified analyses by maternal age (< 29.2 or ≥ 29.2 years), pre-pregnancy BMI (< 20.5 or ≥ 20.5 kg/m^2^), primiparity (yes or no), gravidity (< 1 or ≥ 2), regular physical activity (yes or no), and family history of diabetes (yes or no). No significant modifications were observed between dietary acid load and GDM risk (all *P*_*interaction*_ > 0.05) (Supplementary Table 1). In sensitivity analyses that excluded participants with age >30 years, abnormal pregnancy BMI, smoking, and alcohol consumption, the results remained stable and the significantly positive associations were still observed (Supplementary Table 2).

## Discussion

In this study of pregnant Chinese women, we found that higher dietary acid load (as reflected by three different dietary acid load indices) in early pregnancy was associated with an increased risk of GDM, even after adjustment for characteristics, lifestyle, and other dietary factors (carbohydrate, dietary fiber, cholesterol, vitamin A, vitamin C, vitamin E, SFAs, and MUFAs). In addition, the positive associations tended to be stronger in women with pre-pregnancy BMI ≥ 20.5 kg/m^2^, primiparity, gravidity ≥ 2, lack of regular physical activity, and having a family history of diabetes. After controlling for potential covariates, FBG, 1-h PBG, and 2-h PBG were all significantly increased in the highest tertile of PRAL scores compared to the lowest tertile. Also, higher NEAP scores and A:P ratio increased 1-h PBG and 2-h PBG, but not FBG levels.

In recent years, the interest of research has focused on the relationship between diet-induced acid load and type 2 diabetes and insulin resistance (12, 13, 17, 32). A prospective cohort study of 66,485 French women and the pooled results from three prospective cohort studies in the United States both showed that dietary acid load was positively associated with the risk of type 2 diabetes (13, 32). Similarly, the results of a meta-analysis that included 14 studies showed that participants in the highest categories of PRAL and NEAP scores had a 19 and 22% increased risk of developing diabetes, respectively, compared to the lowest categories (17). Another prospective study in a Korean middle-aged and elderly population found that a higher diet-dependent acid load was associated with an increased risk of insulin resistance in the future (12). To our knowledge, however, research investigating the relationship between dietary acid load and the risk of GDM has been limited to date. In line with our findings, a case-control study conducted in Iran reported a positive association between dietary acid load and risk of GDM measured by PRAL score and A:P ratio (23). Furthermore, apart from finding a positive relationship between PRAL scores and FBG as in the previous study (23), we also identified significant relationships of dietary acid load with 1-h PBG and 2-h PBG.

Our study used three different methods to calculate dietary acid load: PRAL, NEAP, and A:P ratio. These methods are calculated based on the intake of protein, phosphorus, potassium, magnesium, and calcium. All these nutrients are acid-base precursors and may be in relation to pH homeostasis in the body (28, 30, 33). Studies have suggested that foods from animals, such as cheese, fish, and meat, have more acid precursors, while fruits and vegetables are net alkalinizing in nature (20, 21, 34). In the current study, the results showed that the PRAL score, NEAP score, and A:P ratio were all strongly positively correlated with the intake of red meat, poultry, fish, and eggs, while there were significant negative correlations with the intake of vegetables, fruits, legumes, and nuts. The findings are consistent with those of previous studies in other populations (32, 35). Notably, previous studies have only confirmed that consumption of single acidic foods (e.g., meat, milk) or alkaline foods (e.g., fruit, vegetables) was associated with the risk of GDM (36–39); however, taking an integrated approach considering the balance of acidic and alkaline foods may be more important than assessing single acidic and alkaline foods.

The underlying mechanisms linking dietary acid load to glucose homeostasis and GDM risk remain to be elucidated. In the current study, we found that individuals in the highest tertile of dietary acid load had higher protein intakes and lower potassium, calcium, and magnesium intakes. Studies have shown that meat and dairy products, as the main sources of animal protein, were significantly associated with a higher risk of GDM (36, 37). Moreover, animal protein and cereal grains have higher contents of sulfur-containing amino acids (methionine, homocysteine, and cysteine), which produce sulfates with acidifying effects during their metabolism and constitute the main contributor to the daily acid load (33, 40). The main food sources of potassium are vegetables and fruits, which also provide other basic cations (e.g., magnesium). A low-potassium diet can lead to the development of impaired glucose tolerance, through impairments in insulin secretion from pancreatic β-cells (41). Also, potassium can involves in acid-base homeostasis by exchanging hydrogen ions across the cell membrane to assist in electroneutrality (42). Low blood pH could reduce the uptake of glucose by muscle tissue, disrupt the binding of insulin to its receptors (43), and further inhibit insulin signaling pathways, which could lead to the development of insulin resistance and diabetes (44). In addition, the high acidity of the diet may stimulate cortisol secretion from the adrenal cortex, and chronically elevated cortisol levels may induce insulin resistance (45, 46). Furthermore, a higher dietary acid load may stimulate the expression of induced NO synthase and increase levels of inflammatory factors, which may in turn be triggers for GDM (47, 48).

The strengths of this study include detailed information on potential confounders and a prospective design, which greatly reduces the chance of reverse causality and provides strong evidence for examining the associations between dietary acid load and GDM risk. Secondly, we used three indicators, PRAL score, NEAP score, and A:P ratio, which could provide a more comprehensive assessment of dietary acid load during pregnancy from different perspectives. In addition, as dietary habits vary considerably between populations, this study provides evidence from the population of Chinese pregnant women, filling a data gap in the relationship between dietary acid load and GDM in this population.

The current study also has some limitations that need to be considered. Firstly, we used the validated FFQ for dietary assessment, which may still be subject to measurement error and inaccuracy. To partially control for reporting bias, we excluded all participants with extreme values of total energy intake (< 500 kcal/day or > 3500 kcal/day) from the analysis and also adjusted food and nutrient intakes according to the energy residual method. Secondly, we only assessed dietary acid load in early pregnancy, whereas subsequent dietary changes in mid and late pregnancy may have some influence on the results. However, diet before the onset of GDM may more accurately reflect the true causal relationship between exposure and outcome, which could exclude the effect of changes to diet after the occurrence of GDM. Finally, we cannot completely exclude the impact of unmeasured residual factors that may influence the association between dietary acid load and GDM risk. However, it is worth noting that our analysis has adjusted for several confounding factors identified in the previous studies including maternal age, education level, pre-pregnancy BMI, primiparity, smoking status, alcohol intake, regular physical activity, and family history of diabetes.

## Conclusion

To our knowledge, this is the first prospective cohort study using a combination of three indicators to assess the association between dietary acid load and the risk of GDM. Collectively, we found that dietary acid load scores in early pregnancy were positively related to GDM risk in Chinese pregnant women. Decreasing dietary acid load may be a preventive strategy to reduce the occurrence of GDM. Underlying biological mechanisms involved in these associations should be identified and further explored in future studies. Besides, further large-scale studies are needed to confirm our findings in other populations.

## Data Availability Statement

The raw data supporting the conclusions of this article will be made available by the authors, without undue reservation.

## Ethics Statement

The studies involving human participants were reviewed and approved by Tongji Medical College of Huazhong University of Science and Technology. The patients/participants provided their written informed consent to participate in this study.

## Author Contributions

LH, GX, and XY contributed to the conceptualization of the study. RZ and YL performed the analysis. SW, LZ, and GL conducted data collection. RZ wrote the manuscript. LH and GX critically revised the manuscript. All authors read and approved the submitted manuscript.

## Conflict of Interest

The authors declare that the research was conducted in the absence of any commercial or financial relationships that could be construed as a potential conflict of interest.

## Publisher’s Note

All claims expressed in this article are solely those of the authors and do not necessarily represent those of their affiliated organizations, or those of the publisher, the editors and the reviewers. Any product that may be evaluated in this article, or claim that may be made by its manufacturer, is not guaranteed or endorsed by the publisher.

## Footnotes

1
http://www.r-project.org

2
http://www.empowerstats.com
