# Supplementary material for: Dietary Acid Load Is Positively Associated With Risk of Gestational Diabetes Mellitus in a Prospective Cohort of Chinese Pregnant Women
Source: Front Nutr. 2022 May 26;9:892698. doi: 10.3389/fnut.2022.892698 (PMC9184257; doi:10.3389/fnut.2022.892698)
Supplement: Supplementary file 1 [file Data_Sheet_1.docx]

Supplementary Material

**Dietary acid load is positively associated with risk of gestational diabetes mellitus in a prospective cohort of Chinese pregnant women**

Rui Zhao^1^, Leilei Zhou^1^, Gang Lei^2^, Shanshan Wang^1^, Yan Li^1^, Xuefeng Yang^1^, Guoping Xiong^2*^and Liping Hao^1*^

^1^Department of Nutrition and Food Hygiene, Hubei Key Laboratory of Food Nutrition and Safety and the Ministry of Education (MOE) Key Laboratory of Environment and Health, School of Public Health, Tongji Medical College, Huazhong University of Science and Technology, Wuhan, Hubei, China

^2^The Central Hospital of Wuhan, Wuhan, Hubei, China

*** Correspondence:**

Liping Hao: haolp@mails.tjmu.edu.cn

Guoping Xiong: Hyh0120@163.com

**Supplementary Table 1.** Subgroup analyses for the associations between maternal dietary acid load and GDM risk among Chinese pregnant women.

**Supplementary Table 2.** Sensitivity analyses for the associations between maternal dietary acid load and GDM risk among Chinese pregnant women.

**Supplementary Table 1.** Subgroup analyses for the associations between maternal dietary acid load and GDM risk among Chinese pregnant women^a^

| **Subgroups** | **N** | **T1** | **T2** | **T3** | ***P*-trend^b^** | ***P* interaction** |
| --- | --- | --- | --- | --- | --- | --- |
| **PRAL score** |  |  |  |  |  |  |
| **Maternal age** |  |  |  |  |  | 0.326 |
| <Median | 659 | 1.00 | **4.81 (2.19, 10.60)** | **3.61 (1.41, 9.27)** | **0.012** |  |
| ≥Median | 668 | 1.00 | 1.15 (0.66, 2.01) | 1.45 (0.77, 2.72) | 0.245 |  |
| **Pre-pregnancy BMI** |  |  |  |  |  | 0.248 |
| <Median | 654 | 1.00 | 1.55 (0.78, 3.11) | 1.54 (0.67, 3.55) | 0.316 |  |
| ≥Median | 673 | 1.00 | **2.55 (1.42, 4.58)** | **2.93 (1.48, 5.78)** | **0.003** |  |
| **Primiparity** |  |  |  |  |  | 0.547 |
| Yes | 1069 | 1.00 | **2.07 (1.26, 3.40)** | **2.21 (1.23, 3.95)** | **0.010** |  |
| No | 258 | 1.00 | 1.74 (0.60, 5.03) | 1.89 (0.55, 6.56) | 0.313 |  |
| **Gravidity** |  |  |  |  |  | 0.551 |
| 1 | 789 | 1.00 | **1.92 (1.06, 3.50)** | 1.90 (0.92, 3.89) | 0.096 |  |
| ≥2 | 538 | 1.00 | 1.93 (0.96, 3.89) | **2.44 (1.12, 5.35)** | **0.027** |  |
| **Regular physical activity** |  |  |  |  |  | 0.492 |
| Yes | 503 | 1.00 | 1.40 (0.73, 2.69) | 1.71 (0.77, 3.80) | 0.189 |  |
| No | 824 | 1.00 | **2.71 (1.45, 5.06)** | **2.63 (1.29, 5.33)** | **0.012** |  |
| **Family history of diabetes** | |  |  |  |  | 0.576 |
| Yes | 150 | 1.00 | **5.86 (1.29, 26.73)** | **5.48 (1.14, 26.25)** | 0.059 |  |
| No | 1165 | 1.00 | **2.04 (1.27, 3.29)** | **2.10 (1.19, 3.71)** | **0.012** |  |
| **NEAP score** |  |  |  |  |  |  |
| **Maternal age** |  |  |  |  |  | 0.733 |
| <Median | 659 | 1.00 | 3.81 (1.80, 8.05) | 2.29 (0.91, 5.76) | 0.152 |  |
| ≥Median | 668 | 1.00 | 1.32 (0.77, 2.28) | 1.55 (0.85, 2.85) | 0.164 |  |
| **Pre-pregnancy BMI** |  |  |  |  |  | 0.522 |
| <Median | 654 | 1.00 | **2.31 (1.15, 4.65)** | 1.89 (0.81, 4.42) | 0.205 |  |
| ≥Median | 673 | 1.00 | **1.86 (1.07, 3.23)** | **2.15 (1.13, 4.10)** | 0.028 |  |
| **Primiparity** |  |  |  |  |  | 0.694 |
| Yes | 1069 | 1.00 | **2.05 (1.27, 3.33)** | **2.00 (1.14, 3.53)** | **0.031** |  |
| No | 258 | 1.00 | 1.71 (0.62, 4.75) | 1.94 (0.58, 6.43) | 0.289 |  |
| **Gravidity** |  |  |  |  |  | 0.588 |
| 1 | 789 | 1.00 | **1.79 (1.00, 3.19)** | 1.85 (0.94, 3.65) | 0.106 |  |
| ≥2 | 538 | 1.00 | **2.32 (1.18, 4.55)** | **2.37 (1.08, 5.21)** | **0.046** |  |
| **Regular physical activity** |  |  |  |  |  | 0.565 |
| Yes | 503 | 1.00 | 1.43 (0.76, 2.69) | 1.49 (0.67, 3.30) | 0.345 |  |
| No | 824 | 1.00 | **2.48 (1.36, 4.54)** | **2.26 (1.15, 4.44)** | **0.040** |  |
| **Family history of diabetes** | |  |  |  |  | 0.207 |
| Yes | 150 | 1.00 | **6.78 (1.39, 33.05)** | **8.00 (1.55, 41.31)** | **0.032** |  |
| No | 1165 | 1.00 | **1.92 (1.22, 3.03)** | **1.75 (1.01, 3.03)** | 0.065 |  |
| **A:P ratio** |  |  |  |  |  |  |
| **Maternal age** |  |  |  |  |  | 0.241 |
| <Median | 659 | 1.00 | **2.28 (1.10, 4.72)** | **3.01 (1.31, 6.94)** | **0.015** |  |
| ≥Median | 668 | 1.00 | 1.57 (0.92, 2.69) | 1.59 (0.87, 2.92) | 0.190 |  |
| **Pre-pregnancy BMI** |  |  |  |  |  | 0.410 |
| <Median | 654 | 1.00 | 1.48 (0.76, 2.89) | 1.68 (0.78, 3.63) | 0.209 |  |
| ≥Median | 673 | 1.00 | **2.24 (1.27, 3.95)** | **2.47 (1.29, 4.71)** | **0.017** |  |
| **Primiparity** |  |  |  |  |  | 0.440 |
| Yes | 1069 | 1.00 | **1.90 (1.17, 3.07)** | **2.13 (1.23, 3.67)** | **0.014** |  |
| No | 258 | 1.00 | 1.64 (0.58, 4.60) | 1.76 (0.54, 5.71) | 0.392 |  |
| **Gravidity** |  |  |  |  |  | 0.473 |
| 1 | 789 | 1.00 | 1.48 (0.84, 2.62) | 1.34 (0.70, 2.57) | 0.516 |  |
| ≥2 | 538 | 1.00 | **2.20 (1.12, 4.33)** | **3.23 (1.51, 6.89)** | **0.003** |  |
| **Regular physical activity** |  |  |  |  |  | 0.716 |
| Yes | 503 | 1.00 | 1.76 (0.94, 3.32) | 1.72 (0.81, 3.66) | 0.227 |  |
| No | 824 | 1.00 | **1.80 (1.00, 3.24)** | **2.16 (1.14, 4.11)** | **0.033** |  |
| **Family history of diabetes** | |  |  |  |  | 0.530 |
| Yes | 150 | 1.00 | **10.86 (2.18, 53.97)** | **8.24 (1.51, 44.89)** | 0.069 |  |
| No | 1165 | 1.00 | **1.60 (1.01, 2.53)** | **1.98 (1.17, 3.34)** | **0.015** |  |

^a^Multivariate-adjusted model was adjusted for maternal age, pre-pregnancy BMI, education, primiparity, smoking status, alcohol intake, regular physical activity, family history of diabetes, intake of carbohydrate, dietary fiber, cholesterol, vitamin A, vitamin C, vitamin E, SFAs, and MUFAs.

^b^Tests for linear trend were conducted by using the median value for each tertile and treating it as a continuous variable in the logistic regression.

Abbreviation: A:P ratio, animal protein to potassium ratio; pre-pregnancy BMI, pre-pregnancy body mass index; GDM, gestational diabetes mellitus; NEAP, net endogenous acid production; PRAL, potential renal acid load; T, tertile.

**Supplementary Table** **2.** Sensitivity analyses for the associations between maternal dietary acid load and GDM risk among Chinese pregnant women^a^

|  | **Tertiles of dietary acid load** | | | ***P-*trend^b^** |
| --- | --- | --- | --- | --- |
|  | **T1** | **T2** | **T3** |  |
| **Excluding maternal age >30 year (n = 822)** | | | | |
| PRAL score | 1.00 | 3.98 (2.03, 7.77) | 3.58 (1.64, 7.83) | 0.003 |
| NEAP score | 1.00 | 3.18 (1.67, 6.03) | 2.58 (1.21, 5.51) | 0.046 |
| A:P ratio | 1.00 | 1.93 (1.06, 3.54) | 2.26 (1.14, 4.47) | 0.034 |
| **Excluding abnormal pre-pregnancy BMI (n = 896)** | | | | |
| PRAL score | 1.00 | 2.02 (1.18, 3.49) | 2.64 (1.41, 4.95) | 0.003 |
| NEAP score | 1.00 | 1.90 (1.13, 3.19) | 1.93 (1.06, 3.50) | 0.048 |
| A:P ratio | 1.00 | 1.93 (1.16, 3.24) | 1.96 (1.09, 3.50) | 0.048 |
| **Excluding alcoholic drinkers before pregnancy (n = 1298)** | | | | |
| PRAL score | 1.00 | 1.93 (1.23, 3.01) | 2.12 (1.25, 3.58) | 0.006 |
| NEAP score | 1.00 | 1.90 (1.23, 2.93) | 1.97 (1.18, 3.27) | 0.016 |
| A:P ratio | 1.00 | 1.81 (1.18, 2.79) | 2.02 (1.23, 3.31) | 0.012 |
| **Excluding smokers before pregnancy (n = 1291)** | | | | |
| PRAL score | 1.00 | 2.02 (1.29, 3.16) | 2.14 (1.27, 3.62) | 0.006 |
| NEAP score | 1.00 | 2.00 (1.30, 3.08) | 1.97 (1.18, 3.28) | 0.017 |
| A:P ratio | 1.00 | 1.70 (1.11, 2.62) | 1.96 (1.20, 3.19) | 0.013 |

^a^Multivariate-adjusted model was adjusted for maternal age, pre-pregnancy BMI, education, primiparity, smoking status, alcohol intake, regular physical activity, family history of diabetes, intake of carbohydrate, dietary fiber, cholesterol, vitamin A, vitamin C, vitamin E, SFAs, and MUFAs.

^b^Tests for linear trend were conducted by using the median value for each tertile and treating it as a continuous variable in the logistic regression.

Abbreviation: A:P ratio, animal protein to potassium ratio; pre-pregnancy BMI, pre-pregnancy body mass index; GDM, gestational diabetes mellitus; NEAP, net endogenous acid production; PRAL, potential renal acid load; T, tertile.
